# Physician Practice Patterns Associated with Diagnostic Evaluation of Patients with Suspected Mild Cognitive Impairment and Alzheimer's Disease

**DOI:** 10.1155/2019/4942562

**Published:** 2019-02-27

**Authors:** Davneet Judge, Jenna Roberts, Rezaul Karim Khandker, Baishali Ambegaonkar, Christopher M. Black

**Affiliations:** ^1^Adelphi Real World, Grimshaw Lane, Cheshire SK10 5JB, UK; ^2^Merck & Co., Inc., 2000 Galloping Hill Road, Kenilworth, New Jersey 07033, USA

## Abstract

The diagnostic process for patients presenting with cognitive decline and suspected dementia is complex. Physicians face challenges distinguishing between normal aging, mild cognitive impairment, Alzheimer's disease, and other dementias. Although there is some evidence for improving attitudes towards the importance of prompt diagnosis, there is limited information describing how physicians approach this diagnostic challenge in practice. This was explored in the present study. Across-sectional survey of primary care and specialist physicians, in 5 European countries, Canada, and the United States, was conducted. Participants were asked about their use of cognitive screening tools and diagnostic technologies, as well as the rationales and barriers for use. In total, 1365 physicians participated in the survey, 63% of whom were specialists. Most physicians stated they use objective cognitive tools to aid the early detection of suspected mild cognitive impairment or Alzheimer's disease in patients. The Mini-Mental State Examination was the most common tool used for initial screening; respondents cited speed and ease of use but noted its lack of specificity. Cerebrospinal fluid biomarker and amyloid positron emission tomography tests, respectively, had been used by only 26% and 32% of physicians in the preceding 6 months, although patterns of use varied across countries. The most commonly cited reasons for not ordering such tests were invasiveness (for cerebrospinal fluid biomarker testing) and cost (for amyloid positron emission tomography imaging). Data reported by physicians reveal differences in the approaches to the diagnostics process in Alzheimer's. A higher proportion of primary care physicians in the United States are routinely incorporating cognitive assessment tools into annual visits, but this is due to country differences in clinical practice. The value of screening tools and regular use could be discussed further with physicians; however, lack of specificity associated with cognitive tools and the investment required from patients and the healthcare system are limiting factors.

## 1. Introduction

Currently, the diagnostic process for a person with suspected cognitive decline is complex. Simple and specific diagnostic tests for dementing illnesses, such as Alzheimer's disease (AD), are not yet available, and patients present with widely varying symptoms [1, 2]. Furthermore the diagnosis of dementing illness in general and of specific forms of dementia in particular often depends on the exclusion of other possible causes of the patient's symptoms. Because of this complexity and because of varying perceptions about the benefits of diagnosis, there is wide variation in the diagnostic process across different countries and across different healthcare providers [3–5].

Typically, the first stage to the identification of dementia is cognitive screening, conducted within primary care. Initial assessment of a patient should include a detailed history from the patient and the main carer, if possible, initial investigation, and a brief cognitive assessment, before referral to secondary or specialist care for further diagnostic testing. The cognitive assessment should have a particular focus on the identification of cognitive function disturbances and any observed impact on activities of daily living [6]. The Mini-Mental State Examination (MMSE) has traditionally been recommended as the brief cognitive assessment tool of choice [7]. Although cognitive screening has been shown to increase case identification [8], the value of performing these has been questioned due to the lack of data demonstrating improved patient outcomes following dementia diagnosis via screening and the limited medical therapies available after diagnosis [9]. However, early detection of dementia, facilitated by screening, provides the opportunity for further diagnostics and a comprehensive management and treatment plan personalised for the patient. The potential benefits of diagnosis for caregivers are also important to recognize, including facilitating planning for the future and access to appropriate information and community support. Spouses have also rated simply understanding what is wrong with their partners as being of high importance [10].

Alzheimer's disease (AD) is the most common form of dementia and has received significant attention with regard to diagnostic criteria. A definitive diagnosis of AD requires pathologic examination of brain tissue at autopsy [11]. However, in the last decade, at least two groups have developed revised criteria, which allow the diagnosis of AD in living persons with an acceptable degree of certainty. The International Work Group (IWG) published new recommendations in 2007 and a revision in 2010 recognizing that AD is a continuous progressive disease that begins many years before clinical symptoms can be observed [12, 13]. Notably, the IWG criteria called for the use of at least one biomarker of AD pathology to support the diagnosis [14]. The National Institute on Aging (NIA) and the Alzheimer's Association (AA) also worked collaboratively to develop another set of diagnostic criteria encompassing asymptomatic and dementia phases of AD [15–17]. The two sets of guidelines differ in how they define the early symptomatic predementia stage of AD. Specifically, the NIA-AA guidelines distinguish between (1) nonspecific mild cognitive impairment (MCI), which can be clinically diagnosed without the use of biomarkers, and (2) MCI due to AD, which requires biomarker testing and is considered a “research” diagnosis. In contrast, the IWG guidelines specifically incorporate biomarker testing into the definition of prodromal AD, in which symptomatic (but nondemented) patients have biomarker evidence of AD pathology [18].

It should be noted that the IWG and NIA-AA documents discussed are appropriately viewed as* criteria for specific diagnoses* rather than recommendations about how to evaluate patients with evidence of cognitive decline. Other prominent guidelines that more thoroughly discuss the diagnostic process—such as those issued by the European Federation of Neurological Societies-European Neurological Society (EFNS-ENS) and the American Academy of Neurology (AAN)—do not recommend* routine* use of CSF biomarker tests and functional PET imaging modalities, suggesting instead that use is restricted to cases where there is diagnostic uncertainty [19–22]. Physicians are therefore confronted with conflicting recommendations regarding the use of these tests, and current practices reportedly vary across countries and sites [8, 9, 23]. Also, many biomarker tests are unavailable in community practices, some are invasive, and many are not covered by insurance plans. In addition, standardized biomarker tests and cut-off points have not been established [24]. Thus, many aspects of the diagnostic process still depend on the practitioner's perceptions, clinical judgment, and experience.

With recent advances and still-emerging research on diagnostic biomarkers, and with evolving and somewhat discrepant diagnostic criteria, practitioners diagnosing and treating patients with cognitive complaints operate within a complex and evolving environment. Although there is some evidence for improving attitudes about the importance of prompt diagnosis, there is very little information about how these recent changes have influenced the diagnostic process used in real-world settings and across different countries. Such information is essential for guiding efforts to educate practitioners about the diagnostic process. To address this, we conducted a survey of primary and secondary practitioners across the five European countries, Canada, and the United States with the goal of understanding current clinical practices related to the diagnostic process for patients who present with suspected cognitive decline. Notably, the survey includes specific questions regarding the clinical use of biomarker tests, about which there is very little information. This report focuses on current practice patterns used by physicians during the diagnostic process, and how those patterns differ across countries.

## 2. Materials and Methods

### 2.1. Study Design and Participants

The study was a cross-sectional survey of primary care and specialist physicians who were personally responsible for managing patients with dementia or cognitive decline in Canada, France, Germany, Italy, Spain, the UK, and the US. Specialist physicians eligible for participation were neurologists, geriatricians, psychiatrists, or psychogeriatricians.

To identify potential survey participants, a master list was created in each country containing names of practitioners involved in the diagnosis and management of patients with dementia. The list comprised treating physicians who had volunteered to participate in observational research. The ratios of primary versus specialist physicians in each country were based on local treatment practices; thus, more PCPs were recruited in the US than in the European countries. To be eligible, physicians were required to have evaluated at least 5 (PCPs) or 10 (specialists; 8 in the UK) MCI patients in the month preceding participation. The rationale for this was to ensure that physicians had sufficient recent experience with the patient group of interest to provide meaningful responses to the survey questions. Those meeting the eligibility criteria and who were willing to participate were invited to complete the survey. Participants completed the survey online from October to December 2017.

The survey was designed to capture a broad overview of current diagnostic behaviours as well as attitudes and perceptions associated with current diagnostic practices and future developments in the field. The survey required approximately 30 minutes to complete and participating physicians were remunerated for their time. The full survey is included in the appendices.

### 2.2. Statistical Analysis

Descriptive statistics were calculated using Version 15.0 of the Stata software package (StataCorp LLC, College Station, Texas, USA) and SPSS Data Collection Survey Reporter 7 (SPSS, Inc.). For continuous variables, the respondent base, mean, and range (minimum and maximum values) are reported. For categorical variables, the respondent base, number, and percentage of responses are reported.

## 3. Results

### 3.1. Characteristics of Respondents

In total, 1365 physicians participated. The distribution of respondents across the seven countries is shown in Table 1 according to their medical specialty. Overall, 63% of respondents were specialists (as per an a priori sampling ratio); neurologists made up half of the specialist sample (32%). Office based physicians accounted for 42% of the sample, 27% of physicians were hospital based, and the remaining shared their time between both settings. On average, PCPs and specialists had 60 and 62 patients, respectively, under their care with MCI. Specialists had more patients with AD (average of 88 patients per specialist) under their care than did PCPs (43 patients).

### 3.2. Perceptions

On average physicians responded that 73% of their patients who present with cognitive decline would receive a cognitive test at some point in the diagnostic pathway and 90% responded that they use or recommend cognitive tools for patients with suspected MCI or AD. In addition, 86% of physicians reported that they regularly question older patients about their cognitive function. Survey participants were asked at what patient age they would suspect cognitive decline to be indicative of MCI or AD. On average, physicians suspected MCI in such patients if they were 60.2 years of age older, and they suspected AD if the patients were 66.3 years of age or older. These ages were slightly lower among specialists (59.6 years and 61.3 years, respectively) versus PCPS and on average higher in the UK than in other countries (mean 62 years and 68 years).

In some classifications, amnestic MCI (aMCI) is distinguished from other forms of MCI and patients with amnestic symptoms have a higher risk of progressing to AD than patients who have MCI without amnestic symptoms [25]. In our survey, 63% of specialists differentiated between aMCI and MCI compared with 49% of PCPs. However, this practice varied across countries, with physicians in Spain (71%) and Italy (72%) more likely to differentiate MCI subtypes than physicians in Germany or the UK (46% each). The differences between PCPs and specialists also varied across countries; in the UK, a lower percentage of PCPs differentiated MCI subtypes compared to specialists (25% vs. 54%). However, in Germany a lower percentage of specialists differentiated vs. PCPs (41% vs. 57%); the low rates of differentiation among specialists in Germany were largely driven by psychiatrists, among whom only 30% differentiated MCI subtypes.

### 3.3. Initial Cognitive Testing

The data demonstrated that 71% of responding physicians would perform initial cognitive testing in patients of any age who had cognitive complaints. Other situations in which physicians would perform initial cognitive testing were patients who had a family history of dementia (29% of physician respondents), patients who were known carriers of a high-risk ApoE4 allele (13%), and patients older than a certain age with no cognitive complaints (10%). PCPs in the US were more likely than PCPs in other countries to perform routine cognitive testing as part of an annual wellness visit or healthcare check (25% versus 8%), possibly because such screening has been considered a standard part of the Medicare annual wellness check since 2011.

**Figure 1:**
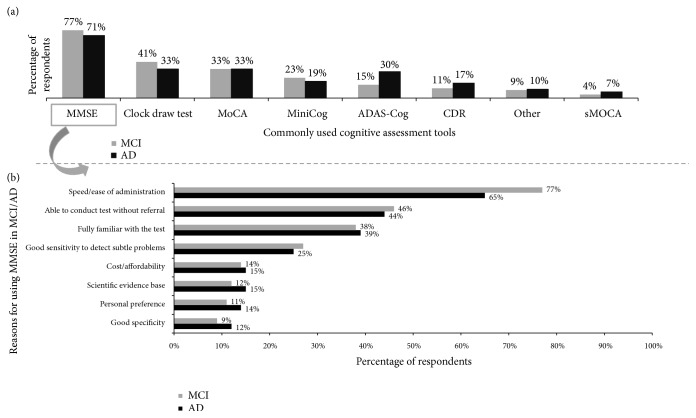
Initial tests of cognitive function commonly used by physician respondents (a) and the most commonly cited reasons for choosing the MMSE among respondents who commonly use it (b).

The physician respondents were asked to select from a list of cognitive assessment tools (including Alzheimer's Disease Assessment Scale–Cognitive subscale (ADAS-Cog), Clinical Dementia Rating (CDR), Clock draw test, MiniCog, Mini-Mental State Examination (MMSE), Montreal Cognitive Assessment (MoCA), and short MoCA (sMoCA)) they commonly used for initial evaluation of patients with suspected cognitive decline. Multiple responses were allowed and the survey permitted physicians to enter any cognitive tool that they used. The results are shown in Figure 1(a). Although there was some variation across countries and medical specialties, the Mini-Mental State Exam (MMSE) was the most commonly identified tool in each country and across all specialties. (In Canada, the Montreal Cognitive Assessment [MoCA] was identified at rates similar to the MMSE.) Other popular tools are shown in Figure 1 but of note, included under the “other” response option, 31 different cognitive instruments were listed by at least one physician, demonstrating huge variation and inconsistency. The physicians who used the MMSE were asked to provide their top 3 reasons for selecting the tool, and the results are shown in Figure 1(b). The speed and ease of administration of the MMSE were identified most frequently in every country and across all medical specialties. Other commonly identified reasons were the ability to conduct the test without referral, familiarity with the tool, and good sensitivity to detect subtle problems. Despite the common use of initial cognitive tools, physicians recognize important barriers to their use such as the time required to administer the tools and their lack of specificity.

### 3.4. Referral for Specialty Care

**Figure 2:**
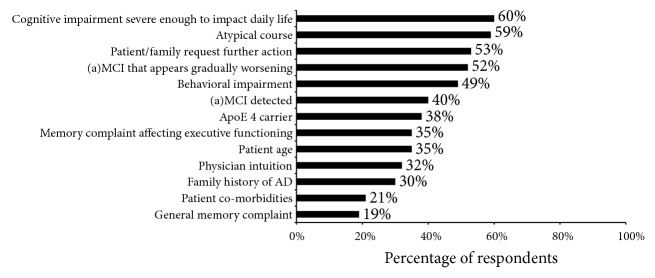
Rationale given by primary care respondents for referral to specialist care.

PCPs were asked to identify situations in which they would refer a patient to a specialist. Multiple responses were accepted and the physicians identified, on average, 5 such situations. These situations are shown in Figure 2 according to how often they were identified by survey respondents. Overall, the most commonly identified situation was cognitive impairment severe enough to impact daily life (60%), but an atypical course was identified nearly as often (59%). Overall, PCPs in the US reported substantially different patterns of referral than other countries; cognitive impairment severe enough to impact daily life was the third most common situation identified (49%) after an atypical course (56%) and request for further action by a family member or companion (53%). Atypical course was also the most commonly identified reason for referral in Canada (72%), and Canadian physicians were also more likely than physicians in other countries to identify signs of worsening disease (58% versus 52% in the 6 other countries) and behavioural impairment (56% versus 48%) as reasons for referral. In France, the most commonly cited reason for referral was behavioural impairment (65%).

### 3.5. Biomarker Testing and Brain Imaging

**Figure 3:**
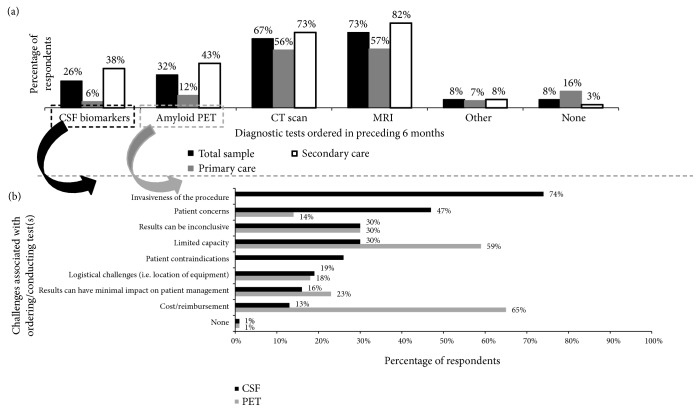
Percentage of respondents ordering diagnostic tests in the preceding 6 months (a). Challenges identified by respondents related to the use of CSF biomarker tests and amyloid PET imaging (b).

The percentages of physician respondents who ordered biomarker and brain imaging tests within the preceding 6 months are shown in Figure 3(a). As expected, CT scans and MRIs were the most commonly ordered tests to evaluate cognitive health (67% and 73% of respondents, respectively), within the 6 months preceding the survey and 26% of physicians had ordered a CSF biomarker test (involving lumbar puncture) in the preceding 6 months (Figure 3). The respondents estimated that approximately 15% of patients presenting with cognitive impairment had received a CSF biomarker test at some point. Primary care physicians were much less likely to have ordered CSF biomarker test than were specialists (6% and 38%, respectively). The percentages of physicians ordering CSF biomarker tests in the preceding 6 months were higher in France (41%), Germany (41%), and Spain (33%) than in other countries: Italy (23%), UK (17%), US (15%), and Canada (10%). Physicians indicated that the greatest challenge related to ordering a CSF biomarker test was the invasiveness of the procedure, but they also cited patient concerns, limited capacity to test everyone, and inconclusive results (Figure 3(b)).

PET amyloid imaging is also one option for obtaining supportive evidence for a diagnosis of AD according to the IWG criteria, [13] but other appropriate use criteria recommend its use only in situations in which the diagnosis is uncertain or there is an atypical presentation [26]. In our survey, 32% of physicians had ordered PET amyloid imaging in the preceding 6 months (Figure 3(a)), and it was estimated that 12% of patients who presented with cognitive impairment had received such imaging at some point. As with CSF biomarker tests, primary care physicians were much less likely to order amyloid PET imaging than were specialists (Figure 3(a)) (12% compared to 43%, respectively). Physicians from Italy (49% of respondents) reported significantly higher usage of this modality than the other countries. The most commonly cited challenges related to ordering PET amyloid imaging were cost/lack of reimbursement, limited capacity to test everyone, and inconclusive result (Figure 3(b)). Physicians in the US (85% of respondents) and the UK (80%) were significantly more likely than physicians in other countries to cite cost as a challenge.

Survey respondents were asked what clinical criteria were most likely to lead them to recommend amyloid biomarker testing by CSF analysis or amyloid PET. The two most common responses were ApoE4 carrier 42% of respondents and a family history of AD (39%). These responses were notably higher than other responses; the third most common responses were cognitive impairment that interferes with daily life and an atypical course (each, 29% of respondents). These findings suggest that decisions to use these biomarker tests may be more strongly influenced by genetic factors in comparison to other diagnostic decisions.

The survey also explored where CSF biomarker and amyloid PET tests were performed, as well as delays between when tests were ordered, when they were conducted, and when the results were available. The majority of physicians performed CSF biomarker tests (66%) within their own practice, although PCPs were more likely to refer patients for testing (60%) versus specialists (32%). On average, results of CSF biomarker tests were available 34.4 days after having been ordered, which included a delay of 14.7 days until sample collection and another 19.7 days until results were available. The country with the shortest average total delay was Germany (22.7 days) and the country with the longest delay was Canada (46.5 days). For amyloid PET imaging, the majority of physicians (69%) referred patients to another provider. On average, results of amyloid PET imaging were available 39.1 days after having been ordered, which included an average delay of 30.6 days before imaging was performed and 8.5 other days until results were available. The country with the shortest average total delay was the US (16.5 days) and the country with the longest delay was Canada (62.7 days).

## 4. Discussion

This survey, conducted in the last months of 2017, sought to probe the current practice patterns of physicians during the process of evaluating patients who present with complaints of cognitive decline. One of the novel aspects of the survey was that it asked physicians about their use of biomarker tests—specifically CSF biomarkers and amyloid PET imaging—during the diagnostic process. Although such tests are discussed in current diagnostic criteria for AD, the tests are relatively new, the data on their use are evolving, and there is not universal agreement about how they should be incorporated into the diagnostic workup. In April 2018 a new framework was published that is intended to guide research towards the goal of defining Alzheimer's disease using biomarkers rather than clinically observed symptoms [27]. It is eagerly anticipated that such a framework will stimulate research leading to universally accepted guidelines on the use of biomarker testing in routine clinical workup. Until that occurs, however, there remain uncertainty and variability regarding the clinical use and reimbursement of biomarker tests for Alzheimer's disease. Accordingly, the physicians in our sample used tests of CSF biomarkers and amyloid PET imaging in only a small percentage of patients. However, amyloid PET imaging was used by substantially more physicians in Italy compared to the other countries in the survey. The reasons for these differences are unclear, but there were also differences across countries in terms of the barriers to the use of these tests. For example, the high cost and challenges around reimbursement of amyloid PET imaging were identified as the biggest challenge to its use in all the countries surveyed, but that barrier was identified by a higher number of physicians in the US and UK than in the countries in which it is used more commonly, such as Italy. CSF biomarker tests are also used in a minority of patients, but the biggest barrier to their use was the invasiveness of the lumbar puncture procedure. Other challenges related to the use of these tests were also identified by survey respondents; for both types of tests, 30% of respondents noted that the results can be inconclusive. An interesting feature of the decision to recommend CSF or amyloid PET biomarker tests was that genetic factors (such as ApoE4 status or a family history of AD) were reported as the strongest factors influencing the decision to use these tests. For other diagnostic decisions, such as the decision to refer to a specialist or to recommend in-depth cognitive testing, these genetic factors ranked as less important than many other factors.

Age alone is generally not sufficient to prompt evaluation of cognitive function; rather, about 70% of respondents indicated that such evaluations are prompted by cognitive complaints. In those cases, a brief cognitive test is a common part of the initial diagnostic workup of an older patient who presents with cognitive complaints, and 70% of physicians in the survey indicated that they would use one of the available brief cognitive tests to help evaluate an older patient with such complaints. Furthermore, a large majority of physicians (90%) in the survey regularly ask older patients about their cognitive function, and cognitive impairment that interferes with daily life was the top driver of both referral to a specialist and in-depth cognitive testing. However, routine use of objective tests at an annual visit in asymptomatic patients was uncommon (<8% of respondents overall), although it was more common in the US (30%). In that country, direct observation of cognitive function became a “required” part of the annual Medicare wellness visit in 2011, and symptoms or concerns should prompt the use of an initial cognitive test.

The most commonly chosen cognitive tool in our survey was the MMSE, for its speed and ease of administration. These responses are consistent with prior studies showing that time constraints and lack of reimbursement for the time required for testing are major factors contributing to inadequate diagnostic evaluation of older patients with cognitive complaints [3, 25, 27]. However, the MMSE is known to lack sensitivity at the earlier stages of dementia so it is unlikely to be an appropriate screening tool. Furthermore, the controversial assertion of copyrights for use of the MMSE means that clinicians face costs for using this test in clinical practice and the risk of legal action for any infringement [28].

As an alternative idea, some authors have advocated the use of simple behavioural indices as a screening tool, such as the “attend alone sign”; i.e., if the patient attends the clinic alone, this is a predictor of dementia being excluded as the cause of any cognitive impairment [26], or the “head-turning sign”; i.e., if the patient turns to the caregiver for help when questioned on something beyond their capacity, this has been shown to be a common feature among patients with dementia [29]. Further research is required to determine the ideal approach for case detection and the specificity and reliability of these indices for patients at different stages of dementia, particularly prodromal stages. However, consensus is emerging on the need for something that is quick, efficient, and cost effective.

*Limitations of the Study. *The survey included a reasonable sample size overall, but the number of physician respondents in specific countries and within specific specialties was limited, so differences across countries and across specialties should be interpreted cautiously. In addition, participants in the survey had previously indicated their interest in observational research and were selected on the basis of high numbers of MCI and Alzheimer's patients in their practices. Therefore, the results may represent more experienced and more engaged practitioners relative to the entire spectrum of community practitioners. Of those who did participate, as with any survey, response and recall bias may apply and there was no objective testing of diagnostic practice and the impact on patient outcomes in this descriptive study. Also, use of many diagnostic practices may depend on a physician's knowledge of current guidelines, which the survey did not query. Thus, the survey results may not provide full explanations about the rationale for specific diagnostic choices.

## 5. Conclusions

Majority of physicians do employ the use of initial screening assessments for their suspected dementia patients and those that are at risk of developing the disease. Whilst physicians appear to be willing to use cognitive tools in the early identification of dementia cases, the limitations of these assessments are well noted among healthcare professionals, namely, the lack of specificity associated with them. Ongoing efforts to redefine Alzheimer's disease on the basis of biomarkers may see increased uptake of CSF and amyloid PET to aid diagnosis in future. At this time, however, these tests are deployed in a low percentage of patients. These findings are consistent with most clinical guidelines on diagnosis of cognitive impairment, which recommend such tests only in cases of diagnostic uncertainty. Thus, despite ongoing research efforts to redefine Alzheimer's disease in terms of biomarkers, the clinical applicability is limited at this time. Physicians in different countries and in different settings frequently exhibit different practice patterns related to the diagnostic evaluation of patients with suspected cognitive decline. These observations suggest that there is continued need for updated and harmonized guidelines intended for real-world clinical use by community physicians and for education and training of physicians involved in the diagnosis and management of patients with cognitive impairment.

## Figures and Tables

**Table 1:** Distribution of survey respondents across countries and across medical specialties.

	n (%)						
	US	Canada	France	Germany	Italy	Spain	UK
Total sample	225	140	200	200	200	200	200
*Primary care* ^*∗*^	*150 (67)*	*50 (36)*	*60 (30)*	*60 (30)*	*60 (30)*	*60 (30)*	*60 (30)*
*Secondary care* ^*∗*^	*75 (33)*	*90 (64)*	*140 (70)*	*140 (70)*	*140 (70)*	*140 (70)*	*140 (70)*
Geriatrician	-	17 (12)	30 (15)	11 (6)	22 (11)	10 (5)	35 (18)
Neurologist	75 (33)	26 (19)	64 (32)	81 (41)	73 (37)	91 (46)	25 (13)
Psychiatrist	-	37 (26)	39 (20)	43 (22)	43 (22)	36 (18)	37 (19)
Psychogeriatrician	-	10 (7)	7 (4)	5 (3)	2 (1)	3 (2)	43 (22)
*Practice setting*							
Hospital	13 (6)	25 (18)	104 (52)	43 (22)	59 (30)	56 (28)	73 (37)
Office	157 (70)	54 (39)	60 (30)	122 (61)	60 (30)	61 (31)	63 (32)
Hospital and office	54 (24)	60 (43)	33 (17)	33 (17)	77 (39)	83 (42)	58 (29)
Other	1 (<1)	1 (1)	3 (2)	2 (1)	4 (2)	nil	6 (3)
*Year qualified *							
Before 1979	14 (6)	8 (6)	8 (4)	3 (2)	4 (2)	2 (1)	12 (6)
1979-1991	79 (35)	47 (34)	56 (28)	49 (25)	69 (35)	43 (22)	76 (38)
1992-2001	75 (33)	43 (31)	81 (41)	67 (34)	73 (37)	83 (42)	82 (41)
2002-2012	56 (25)	37 (26)	55 (28)	73 (37)	50 (25)	72 (36)	29 (15)
After 2012	1 (<1)	5 (4)	nil	8 (4)	4 (2)	nil	1 (1)

^*∗*^As per an a priori sampling ratio.

## Data Availability

The data used to support the findings of this study are available from the corresponding author upon request.
